# The genome sequence of a woodlouse fly, *Melanophora roralis* (Linnaeus, 1758)

**DOI:** 10.12688/wellcomeopenres.23295.1

**Published:** 2024-10-30

**Authors:** Ryan Mitchell, Olga Sivell, Natural History Museum Genome Acquisition Lab, Darwin Tree of Life Barcoding collective, Wellcome Sanger Institute Tree of Life Management, Samples and Laboratory team, Wellcome Sanger Institute Scientific Operations: Sequencing Operations, Wellcome Sanger Institute Tree of Life Core Informatics team, Tree of Life Core Informatics collective, Darwin Tree of Life Consortium

**Affiliations:** 1 Independent researcher, Sligo, County Sligo, Ireland; 2 Natural History Museum, London, England, UK

**Keywords:** Melanophora roralis, woodlouse fly, genome sequence, chromosomal, Diptera

## Abstract

We present a genome assembly from an individual female woodlouse fly, *Melanophora roralis* (Arthropoda; Insecta; Diptera; Rhinophoridae). The genome sequence has a total length of 565.10 megabases. Most of the assembly (98.9%) is scaffolded into 6 chromosomal pseudomolecules, including the X sex chromosome. The mitochondrial genome has also been assembled and is 19.44 kilobases in length. Gene annotation of this assembly on Ensembl identified 20,321 protein-coding genes.

## Species taxonomy

Eukaryota; Opisthokonta; Metazoa; Eumetazoa; Bilateria; Protostomia; Ecdysozoa; Panarthropoda; Arthropoda; Mandibulata; Pancrustacea; Hexapoda; Insecta; Dicondylia; Pterygota; Neoptera; Endopterygota; Diptera; Brachycera; Muscomorpha; Eremoneura; Cyclorrhapha; Schizophora; Calyptratae; Oestroidea; Rhinophoridae; *Melanophora*; *Melanophora roralis* (Linnaeus, 1758) (NCBI:txid1606788).

## Background

*Melanophora roralis* (Linnaeus, 1758) is a small black fly with characteristic darkened wings, measuring 3.3–5.5 mm ( van Emden, 1954). This species is from the subfamily Rhinophorinae (Diptera, Calliphoridae), commonly called woodlice flies. Worldwide there are four described species assigned to genus *Melanophora*, however only *M. roralis* occurs commonly in Europe ( Cerretti *et al.*, 2020). It is widely distributed in the Palaearctic region except in the northernmost parts; in the Afrotropical region it occurs in Cape Verde; it is also reported from the Nearctic and Neotropical regions, where it was apparently introduced ( Cerretti *et al.*, 2020; Sabrosky & Arnaud, 1965). In Britain and Ireland, *M. roralis* is widely distributed. Adults occur from May to October ( NBN Trust, 2024) and can be found resting on light-coloured vertical surfaces, such as walls or rocks. This species parasitizes woodlice living under bark. Unlike other woodlouse flies, it also targets terrestrial isopods living under stones and among vegetation in upper seashore regions, in outhouses and gardens ( Bedding, 1965; van Emden, 1954).

*Melanophora roralis* is very characteristic with strongly infuscated wings, dark brown calypters and dark halteres. Females have a distinct white patch at the wing tip. Vein R _4+5_ has a long petiole, longer than the postangular section of vein M _1_, costal spine absent. The thorax and abdomen are black and shiny, the legs are black ( van Emden, 1954; Zeegers & van Veen, 1993).

Three larval instars and the pupa of *Melanophora roralis* were described by Thompson (1934); egg and first instar larva were described and illustrated by Bedding ( 1965; 1973). Bedding (1973) provides figures and keys for all larval instars of seven British rhinophorid species ( *Stevenia deceptoria* (Loew, 1847) was not known from Britain at the time).

*Melanophora roralis* larvae are parasitoids of *Porcelio scaber* Latreille, 1804 woodlice ( Bedding, 1965; Bedding, 1973; Sassaman & Garthwaite, 1984; Thompson, 1934). Séguy (1941) lists additional host species: the moth *Asopia farinalis* (Linnaeus, 1758), spider *Epeira cornuta* Menge, 1866 (= *Larinioides cornutus* (Clerck, 1757)) and woodlouse *Oniscus asellus* Linnaeus, 1758 *.* Van Emden (1954) also mentions a record of larvae being predacious in egg-cocoons of the spider *Aranea foliata* Fourcroy, 1785 (= *Larinioides cornutus* (Clerck, 1757)), but does not provide the reference or any further information. In laboratory-based experiments, *M. roralis* was able to complete its life cycle using *Cylisticus convexus* (De Geer, 1778), *Porcellio dilatatus* Brandt, 1831, *Porcellionides floria* Garthwaite & Sassaman, 1985 and *Porcellionides pruinosus* (Brandt, 1833) ( Bedding, 1965; Sassaman & Pratt, 1992).

The life cycle of *M. roralis* was described by Bedding (1965), based on rearing in laboratory and field observations. The females are ready for mating within an hour of emerging from puparia (they do not require a meal for their ovaries to mature), while males usually copulate after twelve hours from eclosion (observed in 25°C, in laboratory conditions). The mating dance observed in the laboratory involves wing fluttering initiated by a female, followed briefly by a male, after which copulation occurs. The female also flutters wings during oviposition. The eggs are laid in batches of four to eight in the cracks of bark contaminated with uropod secretions. Usually, all eggs (between 150 and 450 in total) are laid within 6 hours, with short breaks. The female dies within 1 to 3 days from oviposition. Eggs hatch after 7 days. The first instar larva remains close to the site of hatching, resting on its posterior end with its body perpendicular to the substrate. When disturbed it extends anteriorly and moves in a circular or figure of eight motion searching for a host; it can also move by somersaulting. It will attach itself to any moving object but will only remain attached to a host. The larva is only able to penetrate the intersegmental membrane of a recently moulted woodlouse. The larva feeds internally, killing the host in the process and pupates inside its remains ( Bedding, 1965).

Rhinophorinae are a monophyletic group, which in the past it has been treated as a family ( Cerretti *et al.*, 2020; Rognes, 1997) or placed as a subfamily within the Tachinidae or Calliphoridae. The subfamily status for the group has also been proposed by Yan *et al.* (2021). Based on transcriptomes they placed the Rhinophorinae within the family Calliphoridae, with Ameniinae as a sister group. The sampling for this study was very limited, however, with only two rhinophorid species having been analysed. The genome sequence of *Melanophora roralis* presented here, as well as a genome of *Phyto melanocephala* (Meigen, 1824) ( Mitchell *et al.*, 2023) will aid research into the phylogenies of oestroid lineages.

**Figure 1.**
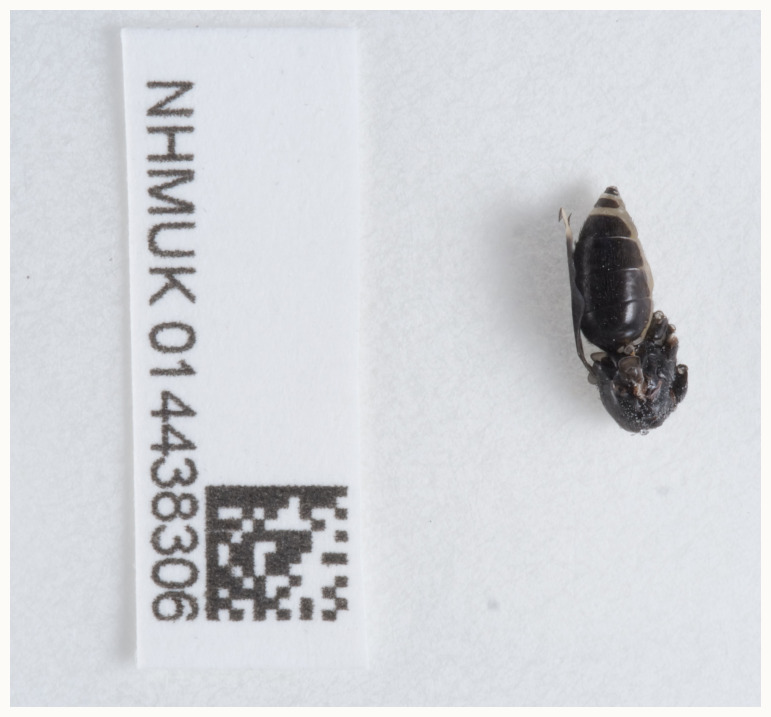
Photograph of the *Melanophora roralis* (idMelRora1) specimen used for genome sequencing.

The high-quality genome of *Melanophora roralis* was sequenced from a single specimen (NHMUK014438306; SAMEA112222163) from Gerrans Bay, England ( Figure 1). The genome was sequenced as part of the Darwin Tree of Life Project, a collaborative effort to sequence all named eukaryotic species in the Atlantic Archipelago of Britain and Ireland.

## Genome sequence report

The genome of *Melanophora roralis* was sequenced using Pacific Biosciences single-molecule HiFi long reads, generating a total of 23.54 Gb (gigabases) from 1.96 million reads, providing approximately 43-fold coverage. Primary assembly contigs were scaffolded with chromosome conformation Hi-C data, which produced 135.96 Gb from 900.39 million reads. Specimen and sequencing details are summarised in Table 1.

**Table 1.** Specimen and sequencing data for *Melanophora roralis*.

Project information			
**Study title**	*Melanophora roralis*		
**Umbrella BioProject**	PRJEB62612		
**Species**	*Melanophora roralis*		
**BioSample**	SAMEA112222163		
**NCBI taxonomy ID**	1606788		
Specimen information			
**Technology**	**ToLID**	**BioSample accession**	**Organism part**
**PacBio long read sequencing**	idMelRora1	SAMEA112222209	Whole organism
**Hi-C sequencing**	idMelRora1	SAMEA112222209	Whole organism
Sequencing information			
**Platform**	**Run accession**	**Read count**	**Base count (Gb)**
**Hi-C Illumina NovaSeq 6000**	ERR11496087	9.00e+08	135.96
**PacBio Sequel IIe**	ERR11483518	1.96e+06	23.54

Assembly errors were corrected by manual curation, including 78 missing joins or mis-joins and 30 haplotypic duplications. This reduced the assembly length by 0.77%, the scaffold number by 42.29%, and the scaffold N50 by 17.83%. The final assembly has a total length of 565.10 Mb in 115 sequence scaffolds, with 336 gaps, and a scaffold N50 of 97.6 Mb ( Table 2).

**Table 2.** Genome assembly data for *Melanophora roralis*, idMelRora1.1.

Genome assembly		
Assembly name	idMelRora1.1	
Assembly accession	GCA_963583895.1	
*Accession of alternate haplotype*	*GCA_963583875.1*	
Span (Mb)	565.10	
Number of contigs	452	
Number of scaffolds	115	
Longest scaffold (Mb)	128.14	
Assembly metrics *		*Benchmark*
Contig N50 length (Mb)	3.9	*≥ 1 Mb*
Scaffold N50 length (Mb)	97.6	*= chromosome N50*
Consensus quality (QV)	61.6	*≥ 40*
*k*-mer completeness	100.0%	*≥ 95%*
BUSCO **	C:98.7%[S:98.1%,D:0.6%], F:0.3%,M:1.0%,n:3,285	*S > 90%* *D < 5%*
Percentage of assembly mapped to chromosomes	98.9%	*≥ 90%*
Sex chromosomes	X	*localised homologous pairs*
Organelles	Mitochondrial genome: 19.44 kb	*complete single alleles*
Genome annotation of assembly GCA_963583895.1 at Ensembl		
Number of protein-coding genes	20,321	
Number of gene transcripts	20,925	

* Assembly metric benchmarks are adapted from Rhie *et al.* (2021) and the Earth BioGenome Project Report on Assembly Standards September 2024. ** BUSCO scores based on the diptera_odb10 BUSCO set using version 5.4.3. C = complete [S = single copy, D = duplicated], F = fragmented, M = missing, n = number of orthologues in comparison.

**Figure 2.**
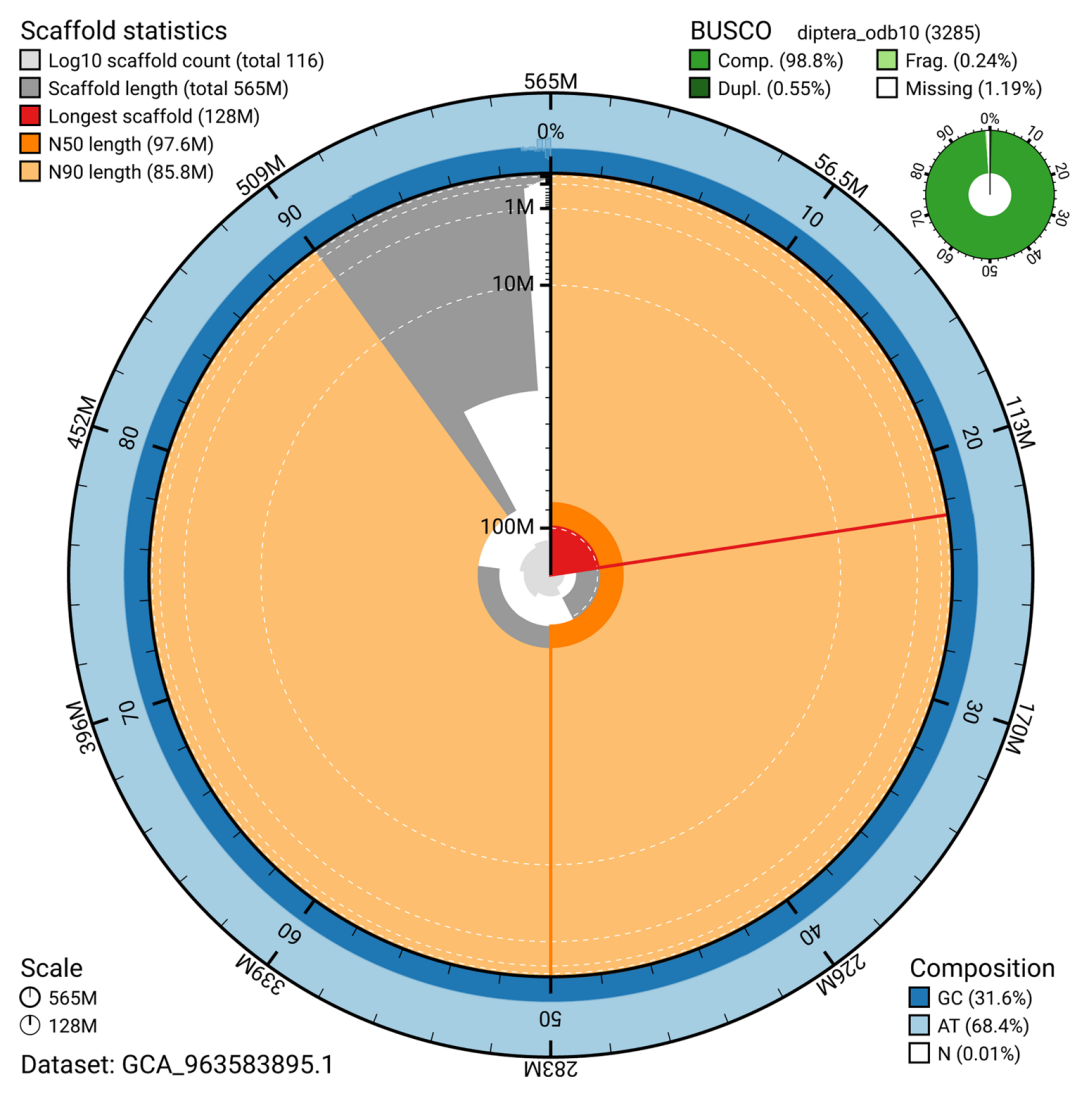
Genome assembly of *Melanophora roralis*, idMelRora1.1: metrics. The BlobToolKit snail plot shows N50 metrics and BUSCO gene completeness. The main plot is divided into 1,000 size-ordered bins around the circumference with each bin representing 0.1% of the 565,136,330 bp assembly. The distribution of scaffold lengths is shown in dark grey with the plot radius scaled to the longest scaffold present in the assembly (128,135,431 bp, shown in red). Orange and pale-orange arcs show the N50 and N90 scaffold lengths (97,632,154 and 85,810,975 bp), respectively. The pale grey spiral shows the cumulative scaffold count on a log scale with white scale lines showing successive orders of magnitude. The blue and pale-blue area around the outside of the plot shows the distribution of GC, AT and N percentages in the same bins as the inner plot. A summary of complete, fragmented, duplicated and missing BUSCO genes in the diptera_odb10 set is shown in the top right. An interactive version of this figure is available at https://blobtoolkit.genomehubs.org/view/GCA_963583895.1/dataset/GCA_963583895.1/snail.

**Figure 3.**
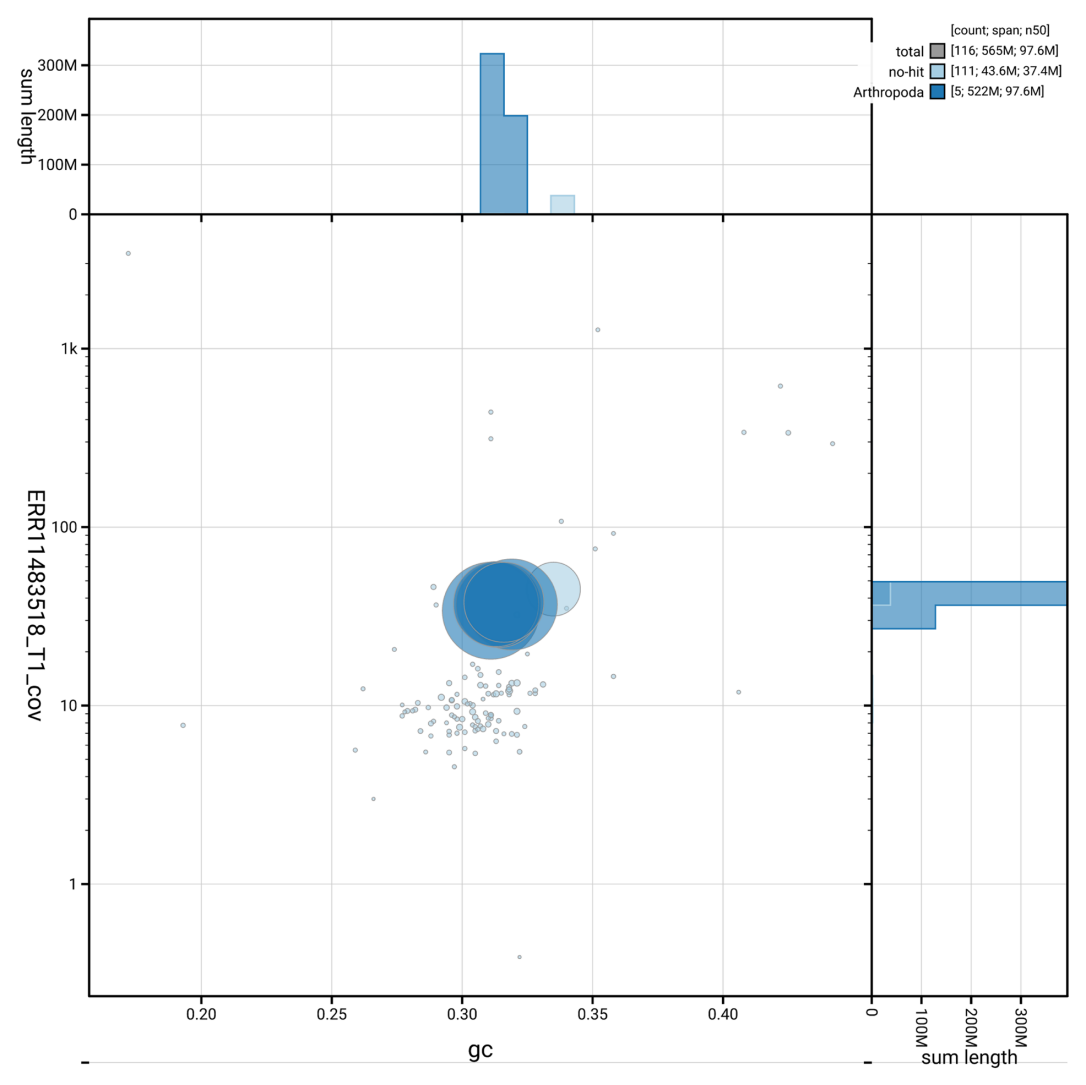
Genome assembly of *Melanophora roralis*: BlobToolKit GC-coverage plot showing sequence coverage (vertical axis) and GC content (horizontal axis). The circles represent scaffolds, with the size proportional to scaffold length and the colour representing phylum membership. The histograms along the axes display the total length of sequences distributed across different levels of coverage and GC content. An interactive version of this figure is available at https://blobtoolkit.genomehubs.org/view/GCA_963583895.1/dataset/GCA_963583895.1/blob.

**Figure 4.**
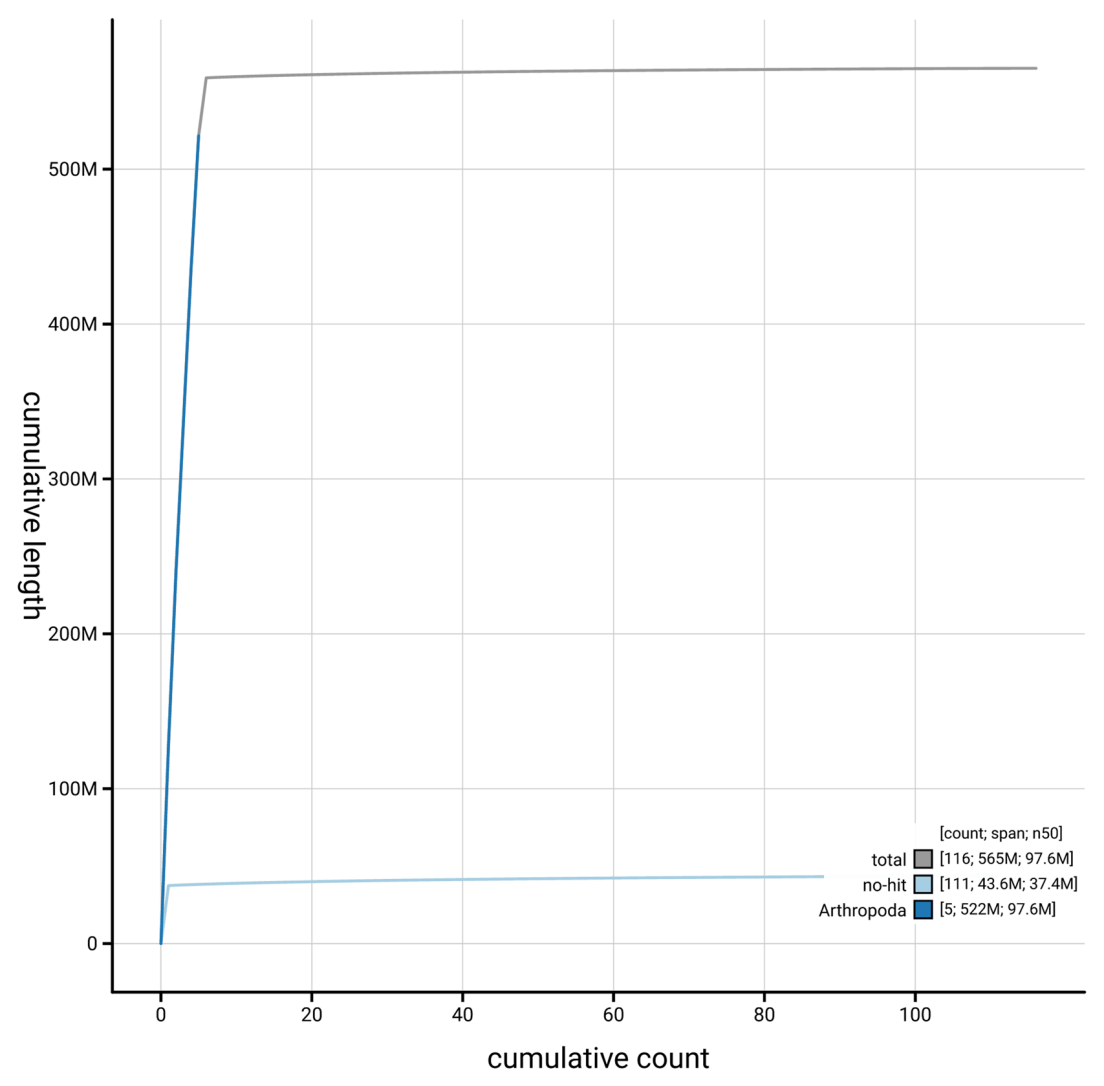
Genome assembly of *Melanophora roralis* idMelRora1.1: BlobToolKit cumulative sequence plot. The grey line shows cumulative length for all scaffolds. Coloured lines show cumulative lengths of scaffolds assigned to each phylum using the buscogenes taxrule. An interactive version of this figure is available at https://blobtoolkit.genomehubs.org/view/GCA_963583895.1/dataset/GCA_963583895.1/cumulative.

The snail plot in Figure 2 provides a summary of the assembly statistics, indicating the distribution of scaffold lengths and other assembly metrics. Figure 3 shows the distribution of scaffolds by GC proportion and coverage, helping to distinguish between various genomic elements. Figure 4 presents a cumulative assembly plot, with separate curves representing different scaffold subsets assigned to various phyla, illustrating the completeness of the assembly.

**Figure 5.**
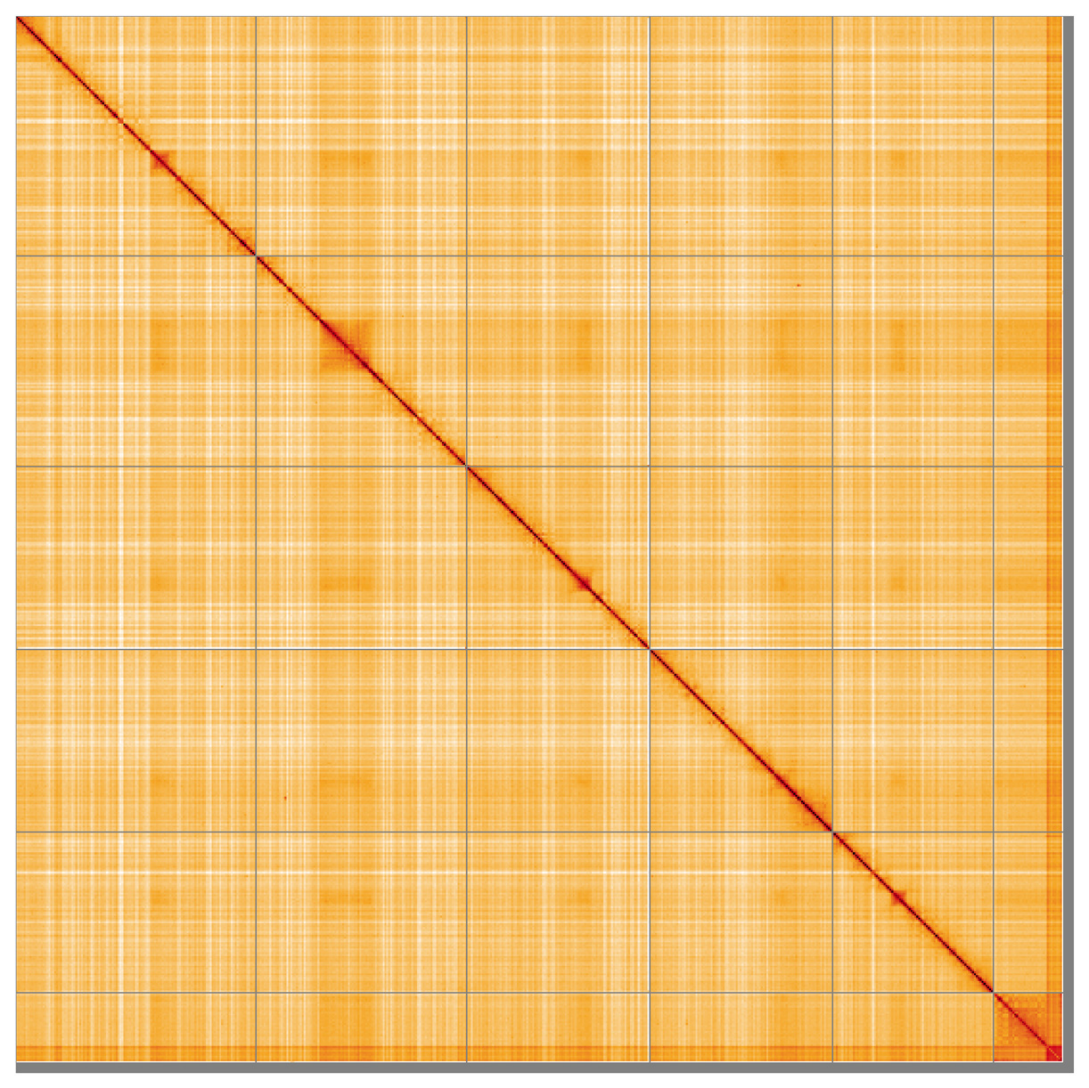
Genome assembly of *Melanophora roralis* idMelRora1.1: Hi-C contact map of the idMelRora1.1 assembly, visualised using HiGlass. Chromosomes are shown in order of size from left to right and top to bottom. An interactive version of this figure may be viewed at https://genome-note-higlass.tol.sanger.ac.uk/l/?d=CWbD9D6wSvWmXBHBkK0qYQ.

Most (98.9%) of the assembly sequence was assigned to 6 chromosomal-level scaffolds, representing 5 autosomes and the X sex chromosome. Chromosome-scale scaffolds confirmed by the Hi-C data are named in order of size ( Figure 5; Table 3). During manual curation, chromosome X was identified by homology with the assembly of *Phyto melanocephala* (GCA_941918925.1) ( Mitchell *et al.*, 2023).

**Table 3.** Chromosomal pseudomolecules in the genome assembly of *Melanophora roralis*, idMelRora1.

INSDC accession	Name	Length (Mb)	GC%
OY757187.1	1	128.14	31.0
OY757188.1	2	112.46	32.0
OY757189.1	3	97.63	31.5
OY757190.1	4	97.47	31.5
OY757191.1	5	85.81	31.5
OY757192.1	X	37.43	33.5
OY757193.1	MT	0.02	17.0

While not fully phased, the assembly deposited is of one haplotype. Contigs corresponding to the second haplotype have also been deposited. The mitochondrial genome was also assembled and can be found as a contig within the multifasta file of the genome submission, and as a separately fasta file with accession OY757193.1.

The final assembly has an estimated Quality Value (QV) of 61.6 and *k*-mer completeness of 100.0%. BUSCO (v5.4.3) analysis using the diptera_odb10 reference set ( *n* = 3,285) indicated a completeness score of 98.7% % (single = 98.1%, duplicated = 0.6%).

Metadata for specimens, BOLD barcode results, spectra estimates, sequencing runs, contaminants and pre-curation assembly statistics are given at https://links.tol.sanger.ac.uk/species/1606788.

## Genome annotation report

The *Melanophora roralis* genome assembly (GCA_963583895.1) was annotated at the European Bioinformatics Institute (EBI) on Ensembl Rapid Release. The resulting annotation includes 20,925 transcribed mRNAs from 20,321 protein-coding genes ( Table 2; https://rapid.ensembl.org/Melanophora_roralis_GCA_963583895.1/Info/Index). The average transcript length is 5,220.78. There are 3.49 exons per transcript.

## Methods

### Sample acquisition and DNA barcoding

A female adult specimen of *Melanophora roralis* (specimen ID NHMUK014438306, ToLID idMelRora1) was collected from Gerrans Bay, England, United Kingdom (latitude 50.2, longitude –4.95) on 2021-06-26, using an aerial net. The specimen was collected and identified by Ryan Mitchell (independent researcher) and preserved by dry freezing at –80°C.

The initial identification was verified by an additional DNA barcoding process according to the framework developed by Twyford *et al*. (2024). A small sample was dissected from the specimens and stored in ethanol, while the remaining parts were shipped on dry ice to the Wellcome Sanger Institute (WSI). The tissue was lysed, the COI marker region was amplified by PCR, and amplicons were sequenced and compared to the BOLD database, confirming the species identification ( Crowley *et al.*, 2023). Following whole genome sequence generation, the relevant DNA barcode region was also used alongside the initial barcoding data for sample tracking at the WSI ( Twyford *et al.*, 2024). The standard operating procedures for Darwin Tree of Life barcoding have been deposited on protocols.io ( Beasley *et al.*, 2023).

### Nucleic acid extraction

The workflow for high molecular weight (HMW) DNA extraction at the Wellcome Sanger Institute (WSI) Tree of Life Core Laboratory includes a sequence of core procedures: sample preparation and homogenisation, DNA extraction, fragmentation and purification. Detailed protocols are available on protocols.io ( Denton *et al.*, 2023b).

The idMelRora1 sample was prepared for DNA extraction by weighing and dissecting it on dry ice ( Jay *et al.*, 2023) and tissue from the whole organism was homogenised using a PowerMasher II tissue disruptor ( Denton *et al.*, 2023a). HMW DNA was extracted using the Automated MagAttract v2 protocol ( Oatley *et al.*, 2023a). DNA was sheared into an average fragment size of 12–20 kb in a Megaruptor 3 system ( Bates *et al.*, 2023). Sheared DNA was purified by solid-phase reversible immobilisation, using AMPure PB beads to eliminate shorter fragments and concentrate the DNA ( Oatley *et al.*, 2023b). The concentration of the sheared and purified DNA was assessed using a Nanodrop spectrophotometer and Qubit Fluorometer using the Qubit dsDNA High Sensitivity Assay kit. Fragment size distribution was evaluated by running the sample on the FemtoPulse system.

### Hi-C preparation

Tissue from the idMelRora1 sample was processed at the WSI Scientific Operations core, using the Arima-HiC v2 kit. In brief, frozen tissue (stored at –80 °C) was fixed, and the DNA crosslinked using a TC buffer with 22% formaldehyde. After crosslinking, the tissue was homogenised using the Diagnocine Power Masher-II and BioMasher-II tubes and pestles. Following the kit manufacturer's instructions, crosslinked DNA was digested using a restriction enzyme master mix. The 5’-overhangs were then filled in and labelled with biotinylated nucleotides and proximally ligated. An overnight incubation was carried out for enzymes to digest remaining proteins and for crosslinks to reverse. A clean up was performed with SPRIselect beads prior to library preparation.

### Library preparation and sequencing

Library preparation and sequencing were performed at the WSI Scientific Operations core. Pacific Biosciences HiFi circular consensus DNA sequencing libraries were prepared using the PacBio Express Template Preparation Kit v2.0 (Pacific Biosciences, California, USA) as per the manufacturer's instructions. The kit includes the reagents required for removal of single-strand overhangs, DNA damage repair, end repair/A-tailing, adapter ligation, and nuclease treatment. Library preparation also included a library purification step using AMPure PB beads (Pacific Biosciences, California, USA) and size selection step to remove templates <3kb using AMPure PB modified SPRI. DNA concentration was quantified using the Qubit Fluorometer v2.0 and Qubit HS Assay Kit and the final library fragment size analysis was carried out using the Agilent Femto Pulse Automated Pulsed Field CE Instrument and gDNA 165kb gDNA and 55kb BAC analysis kit. Samples were sequenced using the Sequel IIe system (Pacific Biosciences, California, USA). The concentration of the library loaded onto the Sequel IIe was between 40–135 pM. The SMRT link software, a PacBio web-based end-to-end workflow manager, was used to set-up and monitor the run, as well as perform primary and secondary analysis of the data upon completion.

For Hi-C library preparation, DNA was fragmented to a size of 400 to 600 bp using a Covaris E220 sonicator. The DNA was then enriched, barcoded, and amplified using the NEBNext Ultra II DNA Library Prep Kit following manufacturers’ instructions. The Hi-C sequencing was performed using paired-end sequencing with a read length of 150 bp on an Illumina NovaSeq 6000 instrument.

### Genome assembly, curation and evaluation

***Assembly***

The HiFi reads were first assembled using Hifiasm ( Cheng *et al.*, 2021) with the --primary option. Haplotypic duplications were identified and removed using purge_dups ( Guan *et al.*, 2020). The Hi-C reads were mapped to the primary contigs using bwa-mem2 ( Vasimuddin *et al.*, 2019). The contigs were further scaffolded using the provided Hi-C data ( Rao *et al.*, 2014) in YaHS ( Zhou *et al.*, 2023) using the --break option. The scaffolded assemblies were evaluated using Gfastats ( Formenti *et al.*, 2022), BUSCO ( Manni *et al.*, 2021) and MERQURY.FK ( Rhie *et al.*, 2020).

The mitochondrial genome was assembled using MitoHiFi ( Uliano-Silva *et al.*, 2023), which runs MitoFinder ( Allio *et al.*, 2020) and uses these annotations to select the final mitochondrial contig and to ensure the general quality of the sequence.

***Assembly curation***

The assembly was decontaminated using the Assembly Screen for Cobionts and Contaminants (ASCC) pipeline (article in preparation). Flat files and maps used in curation were generated in TreeVal ( Pointon *et al.*, 2023). Manual curation was primarily conducted using PretextView ( Harry, 2022), with additional insights provided by JBrowse2 ( Diesh *et al.*, 2023) and HiGlass ( Kerpedjiev *et al.*, 2018). Scaffolds were visually inspected and corrected as described by Howe *et al*. (2021). Any identified contamination, missed joins, and mis-joins were corrected, and duplicate sequences were tagged and removed. The X chromosome was identified by synteny analysis. The curation process is documented at https://gitlab.com/wtsi-grit/rapid-curation (article in preparation).

***Evaluation of the final assembly***

The final assembly was post-processed and evaluated using the three Nextflow ( Di Tommaso *et al.*, 2017) DSL2 pipelines: sanger-tol/readmapping ( Surana *et al.*, 2023a), sanger-tol/genomenote ( Surana *et al.*, 2023b), and sanger-tol/blobtoolkit ( Muffato *et al.*, 2024). The readmapping pipeline aligns the Hi-C reads using bwa-mem2 ( Vasimuddin *et al.*, 2019) and combines the alignment files with SAMtools ( Danecek *et al.*, 2021). The genomenote pipeline converts the Hi-C alignments into a contact map using BEDTools ( Quinlan & Hall, 2010) and the Cooler tool suite ( Abdennur & Mirny, 2020). The contact map is visualised in HiGlass ( Kerpedjiev *et al.*, 2018). This pipeline also generates assembly statistics using the NCBI datasets report ( Sayers *et al.*, 2024), computes *k*-mer completeness and QV consensus quality values with FastK and MERQURY.FK, and runs BUSCO ( Manni *et al.*, 2021) to assess completeness.

The blobtoolkit pipeline is a Nextflow port of the previous Snakemake Blobtoolkit pipeline ( Challis *et al.*, 2020). It aligns the PacBio reads in SAMtools and minimap2 ( Li, 2018) and generates coverage tracks for regions of fixed size. In parallel, it queries the GoaT database ( Challis *et al.*, 2023) to identify all matching BUSCO lineages to run BUSCO ( Manni *et al.*, 2021). For the three domain-level BUSCO lineages, the pipeline aligns the BUSCO genes to the UniProt Reference Proteomes database ( Bateman *et al.*, 2023) with DIAMOND ( Buchfink *et al.*, 2021) blastp. The genome is also split into chunks according to the density of the BUSCO genes from the closest taxonomic lineage, and each chunk is aligned to the UniProt Reference Proteomes database with DIAMOND blastx. Genome sequences without a hit are chunked with seqtk and aligned to the NT database with blastn ( Altschul *et al.*, 1990). The blobtools suite combines all these outputs into a blobdir for visualisation.

The genome assembly and evaluation pipelines were developed using nf-core tooling ( Ewels *et al.*, 2020) and MultiQC ( Ewels *et al.*, 2016), relying on the Conda package manager, the Bioconda initiative ( Grüning *et al.*, 2018), the Biocontainers infrastructure ( da Veiga Leprevost *et al.*, 2017), as well as the Docker ( Merkel, 2014) and Singularity ( Kurtzer *et al.*, 2017) containerisation solutions.

Table 4 contains a list of relevant software tool versions and sources.

**Table 4.** Software tools: versions and sources.

Software tool	Version	Source
BEDTools	2.30.0	https://github.com/arq5x/bedtools2
BLAST	2.14.0	ftp://ftp.ncbi.nlm.nih.gov/blast/executables/blast+/
BlobToolKit	4.3.7	https://github.com/blobtoolkit/blobtoolkit
BUSCO	5.4.3 and 5.5.0	https://gitlab.com/ezlab/busco
bwa-mem2	2.2.1	https://github.com/bwa-mem2/bwa-mem2
Cooler	0.8.11	https://github.com/open2c/cooler
DIAMOND	2.1.8	https://github.com/bbuchfink/diamond
fasta_windows	0.2.4	https://github.com/tolkit/fasta_windows
FastK	427104ea91c78c3b8b8b49f1a7d6bbeaa869ba1c	https://github.com/thegenemyers/FASTK
Gfastats	1.3.6	https://github.com/vgl-hub/gfastats
GoaT CLI	0.2.5	https://github.com/genomehubs/goat-cli
Hifiasm	0.16.1-r375	https://github.com/chhylp123/hifiasm
HiGlass	44086069ee7d4d3f6f3f0012569789ec138f42b84aa44357826c0b6753eb28de	https://github.com/higlass/higlass
Merqury.FK	d00d98157618f4e8d1a9190026b19b471055b22e	https://github.com/thegenemyers/MERQURY.FK
MitoHiFi	3	https://github.com/marcelauliano/MitoHiFi
MultiQC	1.14, 1.17, and 1.18	https://github.com/MultiQC/MultiQC
NCBI Datasets	15.12.0	https://github.com/ncbi/datasets
Nextflow	23.04.0-5857	https://github.com/nextflow-io/nextflow
PretextView	0.2	https://github.com/sanger-tol/PretextView
purge_dups	1.2.5	https://github.com/dfguan/purge_dups
samtools	1.16.1, 1.17, and 1.18	https://github.com/samtools/samtools
sanger-tol/ascc	-	https://github.com/sanger-tol/ascc
sanger-tol/genomenote	1.1.1	https://github.com/sanger-tol/genomenote
sanger-tol/readmapping	1.2.1	https://github.com/sanger-tol/readmapping
Seqtk	1.3	https://github.com/lh3/seqtk
Singularity	3.9.0	https://github.com/sylabs/singularity
TreeVal	1.0.0	https://github.com/sanger-tol/treeval
YaHS	1.2a.2	https://github.com/c-zhou/yahs

### Genome annotation

The BRAKER2 pipeline ( Brůna *et al.*, 2021) was used in the default protein mode to generate annotation for the *Melanophora roralis* assembly (GCA_963583895.1) in Ensembl Rapid Release at the EBI.

### Wellcome Sanger Institute – Legal and Governance

The materials that have contributed to this genome note have been supplied by a Darwin Tree of Life Partner. The submission of materials by a Darwin Tree of Life Partner is subject to the **‘Darwin Tree of Life Project Sampling Code of Practice’**, which can be found in full on the Darwin Tree of Life website here. By agreeing with and signing up to the Sampling Code of Practice, the Darwin Tree of Life Partner agrees they will meet the legal and ethical requirements and standards set out within this document in respect of all samples acquired for, and supplied to, the Darwin Tree of Life Project.

Further, the Wellcome Sanger Institute employs a process whereby due diligence is carried out proportionate to the nature of the materials themselves, and the circumstances under which they have been/are to be collected and provided for use. The purpose of this is to address and mitigate any potential legal and/or ethical implications of receipt and use of the materials as part of the research project, and to ensure that in doing so we align with best practice wherever possible. The overarching areas of consideration are:

•   Ethical review of provenance and sourcing of the material

•   Legality of collection, transfer and use (national and international)

Each transfer of samples is further undertaken according to a Research Collaboration Agreement or Material Transfer Agreement entered into by the Darwin Tree of Life Partner, Genome Research Limited (operating as the Wellcome Sanger Institute), and in some circumstances other Darwin Tree of Life collaborators.

## Data Availability

European Nucleotide Archive: Melanophora roralis. Accession number PRJEB62612; https://identifiers.org/ena.embl/PRJEB62612 ( Wellcome Sanger Institute, 2023). The genome sequence is released openly for reuse. The *Melanophora roralis* genome sequencing initiative is part of the Darwin Tree of Life (DToL) project. All raw sequence data and the assembly have been deposited in INSDC databases. Raw data and assembly accession identifiers are reported in Table 1 and Table 2.

## Author information

Members of the Natural History Museum Genome Acquisition Lab are listed here: https://doi.org/10.5281/zenodo.12159242.

Members of the Darwin Tree of Life Barcoding collective are listed here: https://doi.org/10.5281/zenodo.12158331.

Members of the Wellcome Sanger Institute Tree of Life Management, Samples and Laboratory team are listed here: https://doi.org/10.5281/zenodo.12162482.

Members of Wellcome Sanger Institute Scientific Operations: Sequencing Operations are listed here: https://doi.org/10.5281/zenodo.12165051.

Members of the Wellcome Sanger Institute Tree of Life Core Informatics team are listed here: https://doi.org/10.5281/zenodo.12160324.

Members of the Tree of Life Core Informatics collective are listed here: https://doi.org/10.5281/zenodo.12205391.

Members of the Darwin Tree of Life Consortium are listed here: https://doi.org/10.5281/zenodo.4783558.
