# Supplementary material for: The genome sequence of a woodlouse fly, Melanophora roralis (Linnaeus, 1758)
Source: Wellcome Open Res. 2024 Oct 30;9:637. [Version 1] doi: 10.12688/wellcomeopenres.23295.1 (PMC11628945; doi:10.12688/wellcomeopenres.23295.1)
Supplement: Peer review history

# Reviewer report: Reviewer response for version 1

**DOI:** 10.21956/wellcomeopenres.25677.r111735

**Published:** 2024-12-09

**Authors:** Qingsong Zhou

**Affiliations:** 1 Chinese Academy of Sciences, Beijing, China

The authors present a high-quality genome assembly of the woodlouse fly ( *Melanophora roralis*). The background provided is highly detailed, encompassing information on both the biology and taxonomy of the species.

 The assembled mitochondrial genome measures 19.44 kilobases in length, which is significantly longer than the typical mitochondrial genomes observed in fly species. The authors should consider verifying this finding against reference mitochondrial genomes available in the National Center for Biotechnology Information (NCBI) database.

 Furthermore, the authors should provide a more detailed description of the "Assembly curation" component within the "Methods" section, particularly regarding the statement, "The assembly was decontaminated using the Assembly Screen for Cobionts and Contaminants (ASCC) pipeline (article in preparation).

 In summary, the manuscript is well composed, and the genome assembly is of high quality. Considering the limited genomic resources available for the family Rhinophoridae, this newly sequenced genome is valuable for future studies on evolutionary and other related aspects.

Are sufficient details of methods and materials provided to allow replication by others?

Yes

Is the rationale for creating the dataset(s) clearly described?

Yes

Are the datasets clearly presented in a useable and accessible format?

Yes

Are the protocols appropriate and is the work technically sound?

Yes

Reviewer Expertise:

Entomology, Genomics

I confirm that I have read this submission and believe that I have an appropriate level of expertise to confirm that it is of an acceptable scientific standard.

# Reviewer report: Reviewer response for version 1

**DOI:** 10.21956/wellcomeopenres.25677.r111742

**Published:** 2024-11-26

**Authors:** Charles Plessy

**Affiliations:** 1 Okinawa Institute of Science and Technology, Okinawa, Japan

This paper follows the standard layout of the Darwin Tree of Life Consortium. As I am not a fly biologist I can not comment on the introduction. This said, I enjoyed reading it. All metrics of quality are good in comparison with the current standards.

 I am a bit puzzled by the cloud of dots under the main spot in the BlobToolKit plot (Figure 3); maybe the authors can comment on it? In any case, I do not think it compromises the quality of the assembly.

 The Hi-C contact map in Figure 5 shows no obvious defect. Out of curiosity I aligned the *M. roralis* assembly to other Oestroidae chromosomal assemblies, but none of them has close proximity so it could only confirm that scaffolds were correctly attributed to chromosomes given the conservation of synteny in this clade. This said, this evolutionary distance between *M. roralis* and the nearest chromosomal assemblies highlights that the authors made a good decision in sequencing this animal.

 I looked at the alternative haplotype and found that it is significantly smaller (460 Mb vs 565). Can the authors comment on the possibility that it may be incomplete? Also, if the authors revise the text, maybe they can consider describing their primary assembly as a "pseudo-haplotype" instead of "not fully phased haplotype"?

Are sufficient details of methods and materials provided to allow replication by others?

Yes

Is the rationale for creating the dataset(s) clearly described?

Yes

Are the datasets clearly presented in a useable and accessible format?

Yes

Are the protocols appropriate and is the work technically sound?

Yes

Reviewer Expertise:

Genomics

I confirm that I have read this submission and believe that I have an appropriate level of expertise to confirm that it is of an acceptable scientific standard.
